# Effect of NLRP3 gene knockdown on pyroptosis and ferroptosis in diabetic cardiomyopathy injury

**DOI:** 10.1186/s12872-024-04010-x

**Published:** 2024-07-10

**Authors:** Jiahui Wang, Yuping Li, Lu Li, Huan Liang, Hongwei Ye, Pinfang Kang, Zhenghong Li, Ying Yu, Qin Gao

**Affiliations:** 1 Department of Anatomy, Key Laboratory of Basic and Clinical Cardiovascular Diseases, Bengbu Medical University, Bengbu, 233000 Anhui P.R. China; 2 Department of Physiology, Key Laboratory of Basic and Clinical Cardiovascular Diseases, Bengbu Medical University, Bengbu, 233000 Anhui P.R. China; 3 Key Laboratory of Basic and Clinical Cardiovascular Diseases, Bengbu Medical University, Bengbu, 233000 Anhui P.R. China; 4 Department of Graduate Studies, Department of Cardiology, The First Affiliated Hospital of Bengbu Medical University, Bengbu, 233004 Anhui P.R. China; 5 Department of Cardiology, The First Affiliated Hospital of Bengbu Medical University, Bengbu, 233004 Anhui P.R. China

**Keywords:** Diabetic cardiomyopathy, NLRP3, Mitochondrial reactive oxygen sepsis, Pyroptosis, Ferroptosis

## Abstract

Diabetic cardiomyopathy (DCM) is a chronic disease caused by diabetes mellitus, which is recognized as a worldwide challenging disease. This study aimed to investigate the role and the potential mechanism of knocking down the NACHT-, LRR- and PYD domains-containing protein 3 (NLRP3), an inflammasome associated with onset and progression of various diseases, on high glucose or diabetes -induced cardiac cells pyroptosis and ferroptosis, two regulated non-necrosis cell death modalities discovered recent years. In the present study, both in vivo and in vitro studies were conducted simultaneously. Diabetic rats were induced by 55 mg/kg intraperitoneal injection of streptozotocin (STZ). Following the intraperitoneal injection of MCC950 (10 mg/kg), On the other hand, the DCM model in H9C2 cardiac cells was simulated with 35 mmol/L glucose and a short hairpin RNA vector of NLRP3 were transfected to cells. The results showed that in vivo study, myocardial fibers were loosely arranged and showed inflammatory cell infiltration, mitochondrial cristae were broken and the GSDMD-NT expression was found notably increased in the DM group, while the protein expressions of xCT and GPX4 was significantly decreased, both of which were reversed by MCC950. High glucose reduced the cell viability and ATP level in vitro, accompanied by an increase in LDH release. All of the above indicators were reversed after NLRP3 knockdown compared with the HG treated alone. Moreover, the protein expressions of pyroptosis- and ferroptosis-related fators were significantly decreased or increased, consistent with the results shown by immunofluorescence. Furthermore, the protective effects of NLRP3 knockdown against HG were reversed following the mtROS agonist rotenone (ROT) treatment. In conclusion, inhibition of NLRP3 suppressed DM-induced myocardial injury. Promotion of mitochondrial ROS abolished the protective effect of knockdown NLRP3, and induced the happening of pyroptosis and ferroptosis. These findings may present a novel therapeutic underlying mechanism for clinical diabetes-induced myocardial injury treatment.

**Supplementary Information:**

The online version contains supplementary material available at 10.1186/s12872-024-04010-x.

## Highlights

Knocking down of NLRP3 directly affect the expression of ASC and caspase-1, and protect against HG -induced cardiac cells pyroptosis.

There is a crosstalk between pyroptosis and ferroptosis through NLRP3.

There is an interactive relationship between mtROS and NLRP3. Promoting mtROS not only act as upstream of NLRP3, but also downstream of NLRP3.

**Supplementary Information:**

The online version contains supplementary material available at 10.1186/s12872-024-04010-x.

## Introduction

Diabetes mellitus (DM), caused primarily by defects in the secretion or action of insulin, is a metabolic disease characterized by hyperglycemia and involves multiple pathogenic processes. As one of the serious and non-negligible complications of DM, the prevalence of diabetic cardiomyopathy (DCM) increases in parallel with the increase in DM. Furthermore, DCM has been reported in a variety of studies to be implicated with elevations in advanced glycation end products (AGEs) and collagen-based cardiomyocyte and extracellular matrix stiffness, inflammation and impaired cardiac insulin metabolic signaling, besides the mitochondrial dysfunction and increased oxidative stress [1–4]. A great deal of efforts has been done on the pathophysiology and pathogenesis of DCM. however, the effective prevention of DCM progression remains a challenge.

Chen et al. summarized the different types of cell death in DCM, including but not limited to pyroptosis and ferroptosis, which are both programmed cell death types closely related to inflammation [5]. The NACHT-, LRR- and PYD domains-containing protein 3 (NLRP3) inflammasome, an important component of innate immune system, was widely known to play a key role in promoting pyroptosis. Inhibition of NLRP3 expression has been shown to protect against injury in numberous diseases. NLRP3 deficiency was found to ameliorate renal inflammation and fibrosis in diabetic mice [6]. Knocking down NLRP3 expression also rescued uric acid-induced insulin signaling impairment in both HepG2 cells and L02 cells [7]. In addition, NLRP3 inflammasome (composed of NLRP3, apoptosis-associated speck-like protein containing CARD (ASC) and caspase-1) is a molecular marker and can be activated in DCM [8]. Moreover, inhibition or silencing NLRP3 gene ameliorates DCM in type 2 diabetes rat [9, 10]. However, whether inhibition NLRP3 can directly affect ASC and caspase-1, the other components of NLRP3 inflammasome, and whether play the role on another inflammation related cell death model – ferroptosis, has not yet been reported.

It is widely accepted that NLRP3 inflammasome could be activated by reactive oxygen species (ROS), ionic flux, mitochondrial dysfunction and lysosomal damage [11]. Moreover, mitochondrial ROS (mtROS), the main source of cellular ROS [12], plays a pivotal role in the regulation of ferroptosis as well [13]. In addition, a previous study revealed that ROS scavenger (N-acetyl-cysteine, NAC) prevented endothelial cell pyroptosis [14]. Regulating mtROS has been found to inhibit acetaminophen-induced liver pyroptosis [15]. Another study also supported the point that Mitoquinone, a mitochondria-targeting antioxidant drug, attenuated ferroptosis in neuronal HT22 cells induced by exposure to RSL3 [16]. These findings indicate that inhibition of mtROS reduces the occurrence of pyroptosis and ferroptosis and that NLRP3 is located downstream of mtROS. However, to the best of our knowledge, whether NLRP3 exists upstream of mtROS remains unclear as of today.

So, the present study aimed to investigate the role of NLRP3 knockdown in high glucose (HG)-induced H9C2 cardiac cell pyroptosis and ferroptosis, and to further explore the relationship between NLRP3 and mtROS.

## Materials and methods

### Animal establishment and treatment

A total of 20 four-week male Sprague-Dawley (SD) rats were purchased from Jinan Peng Yue Laboratory Animal Breeding Co.Ltd (Shandong, China) and were given a single intraperitoneal injection with streptozotocin (STZ, Cat No. HY-13,753, MedChemExpress, NJ, USA) at dosage 55 mg/kg after 1–2 weeks of adaptation feeding. Rats with glucose values ≥ 16.7 mmol/L one week after injection were randomized into two groups: Diabetes mellitus (DM) group, and intraperitoneal injection with MCC950 (Cat No. HY-12,815 A, MedChemExpress, NJ, USA), an inhibitor of NLRP3 (DM + MCC950, 10 mg/kg, twice per week) group. Meanwhile, the control group (Sham) and untreated group (DM) received an equal volume of vehicle (saline). The treatment of the three groups lasted for 6 weeks. Body weight (BW) and fasting blood glucose (FBG) levels were measured weekly until sacrifice. And the serum and heart samples were obtained after anesthesia (intraperitoneal injection of pentobarbital sodium at 40 mg/kg). All protocols were carried out according to the approved Institutional Animal Care and Use Committee protocol of Bengbu Medical College (no. 075, 2017).

### H&E staining

*T*he fresh myocardial tissue samples were collected from each group, fixed in 4% paraformaldehyde for 24 h, dehydrated in an ethanol gradient, embedded in paraffin, and the fixed myocardial tissue was sectioned (0.5 mm), stained with hematoxylineosin, and analyzed under light microscopy (Nikon Eclipse E100) for structural changes in myocardial tissue.

### Transmission electron microscopy (TEM)

Briefly, the rat hearts were removed and washed in PBS, then the apical tissue was removed within 3 minutes, 1 mm^3^ apical tissue was immediately placed in centrifuge tubes with 2.5% glutaraldehyde fixative and stored at 4 ˚C. The tissues were fixed in 1% osmium acid for 2 h at room temperature away from light, and ultrathin Sect. (70 nm) were dehydrated at room temperature, osmotically embedded, polymerized, stained, and observed under a transmission electron microscope (HITACHI, HT7800) for ultrastructural images of the myocardium.

### Cell culture and treatment

The H9C2 cardiac cells (Fubio Biological Technology Co., Ltd, Suzhou, China) were cultured in DMEM (Gibco; Thermo Fisher Scientific, MA, USA) supplemented with 10% fetal bovine serum (FBS, Gibco; Thermo Fisher Scientific, MA, USA) and 100 µg/ml penicillin/streptomycin (Beyotime Institute of Biotechnology, Shanghai, China) in a 5% CO_2_ incubator at 37˚C. To generate a high glucose injury model, H9C2 cells were incubated in 35 mmol/L glucose medium for 24 h after 16–18 h of serum-free synchronization treatment. And Rotenone (ROT, Cat No. B5462, APExBIO, SFO, USA), a mitochondrial ROS agonist, dissolved in 35 mmol/l glucose medium at a final concentration of 0.5 µmol/ml was added to the cells after the transfection of small hairpin RNA (shRNA).

### ShRNA transfection

Three target shRNA vectors of NLRP3 (sh-NLRP3#1, #2 and #3) and negative control vector (sh-NC) were designed and synthesized by Hanheng Co., Ltd, Shanghai, China. H9C2 cells were seeded into 6-well plates and then transfected with sh-NLRP3 and sh-NC at a multiplicity of infection of 60 in the presence of 5 µg/ml polybrene according to the manufacturer’s protocols. After 72 h of transfection, infection efficiency was assessed under fluorescence microscopy (magnification, ×100, Olympus, Tokyo, Japan) and successful NLRP3 knockdown cells was evaluated by western blotting.

*Cell Counting Kit-8 (CCK-8) assay.* Approximately 1 × 10^4^ cells were plated into 96-well plates, then 10 µl CCK-8 reagent (Cat No. C0037, Beyotime Institute of Biotechnology, Shanghai, China) was added into plates for 2 h to incubation following groups of treatments. Cell viability was measured using a spectrophotometer (Thermo Fisher Scientific, Inc. MA, USA) with a density at 450 nm.

### Lactate dehydrogenase (LDH) production assay

A LDH assay kit (Cat No. A020-2-2, Nanjing Jiancheng Bioengineering Institute, Nanjing, China) was used to quantify the cytotoxicity. Briefly, the cells were placed in a centrifuge at 4˚C, 600 g for 5 min to collect supernatant. And the serum samples of rats were extracted from centrifuged whole blood at 4˚C, 3000 r for 15 min. Double-distilled water, pyruvate standard, cell supernatants and serum samples, NaOH, and other reagents were then added to the 96-well plate in order and the plate was incubated at 37˚C according to the manufacturer’s instructions. The absorbance values were measured at 450 nm after standing the plate for 5 min.

### Adenosine 5’-triphosphate (ATP) content detection

The cellular ATP levels were measured using a firefly luciferase-based ATP assay kit (Cat No. S0026, Beyotime Institute of Biotechnology, Shanghai, China). Cell samples, collected by placing lysed H9C2 cells in a 4˚C, 12,000 g for 5 min, and ATP assays were added sequentially into a light-proof 96-well plate according to the manufacturer’s protocol. The spectrophotometer was used to detect the ATP concentration and the corresponding protein concentration were detected to reduce errors.

### Western blotting

Total protein was extracted from cells/animal tissues using radioimmunoprecipitation assay lysis buffer combined with 1% phenylmethanesulfonyl fluoride on ice and the concentration was determined with a BCA kit (Cat No. P0012S, Beyotime Institute of Biotechnology, Shanghai, China). A mass of 30 µg of protein was transferred to PVDF membranes and blocked with 5% skimmed milk for 2 h after being separated by 12.5% or 15% SDS-PAGE. Membranes were respectively incubated overnight at 4˚C with primary antibodies of anti-NLRP3 (Cat No. NBP2-12446, 1/600, Novus, CO, USA), anti-ASC (Cat No. ab175449, 1/250, Abcam, Cambridge, UK), anti-Cleaved-caspase-1 (Cat No. AF4005, 1/500, Affinity, Shanghai, China), anti-Cleaved-Gasdermin D (GSDMD-NT, Cat No. 93709s, 1/2,000, Cell Signaling Technology, MA, USA), anti-interleukin-1β (IL-1β) (Cat No. YR0918081, 1/2,000, R&D, MN, USA), anti-IL-18 (Cat No. C2U0215071, 1/2,000, R&D, MN, USA), anti-glutathione peroxidase 4 (GPX4) (Cat No. ab125066, 1/3,000, Abcam, Cambridge, UK), anti-xCT (Cat No. ab175186, 1/3000, Abcam, Cambridge, UK), and anti-GAPDH (Cat No. abs118936a, 1/3,000, Absin, Shanghai, China). Membranes were incubated with the HRP-conjugated secondary antibodies, rabbit anti-goat (Cat No. BA1060, 1/500 − 10,000, BOSTER Biological Technology, Wuhan, China) and goat anti-rabbit (Cat No. Abs20022ss, 1/10,000, Absin, Shanghai, China) for 1–2 h at room temperature. The membranes were visualized with enhanced chemiluminescence reagent (Millipore, ECL System, MA, USA) and Image J software were used to quantize the grayscale values. Protein levels were normalized to GAPDH.

### Immunofluorescence staining

H9C2 cells are seeded at 2 × 10^4^ cells/well into 12-well plates. Following subgroup treatment, 4% paraformaldehyde and 0.5% Triton X-100 were successively used to fix and permeabilize the cells at 37˚C. Then cells were incubated overnight in primary antibodies of anti-NLRP3 (Cat No. NBP2-12446, 1/50, Novus, CO, USA) and anti-caspase-1 Cat No. ab1872, 1/200, Abcam, Cambridge, UK) diluted in BSA after being blocked with BSA for half an hour. The following day, the secondary antibody of CyTM3 (Cat No. 144,930, 1/600, Jackson Immunoresearch Laboratories, PA, USA). Images were observed with an inverted fluorescence microscope (magnification, ×200, Olympus, Tokyo, Japan) and ImageJ software was used to analyze the fluorescence intensity.

### Statistical analysis

All statistical analyses in the present study were performed using SPSS version 24.0 software. ANOVA was conducted to determine variabilities for multiple groups followed by Tukey’s post hoc test and *P* < 0.05 was considered to indicate a statistically significant difference. Data are presented as mean ± SD of at least three independent experiments.

## Results

### Inhibition of NLRP3 reduces injury in diabetic rats

**Fig. 1:**
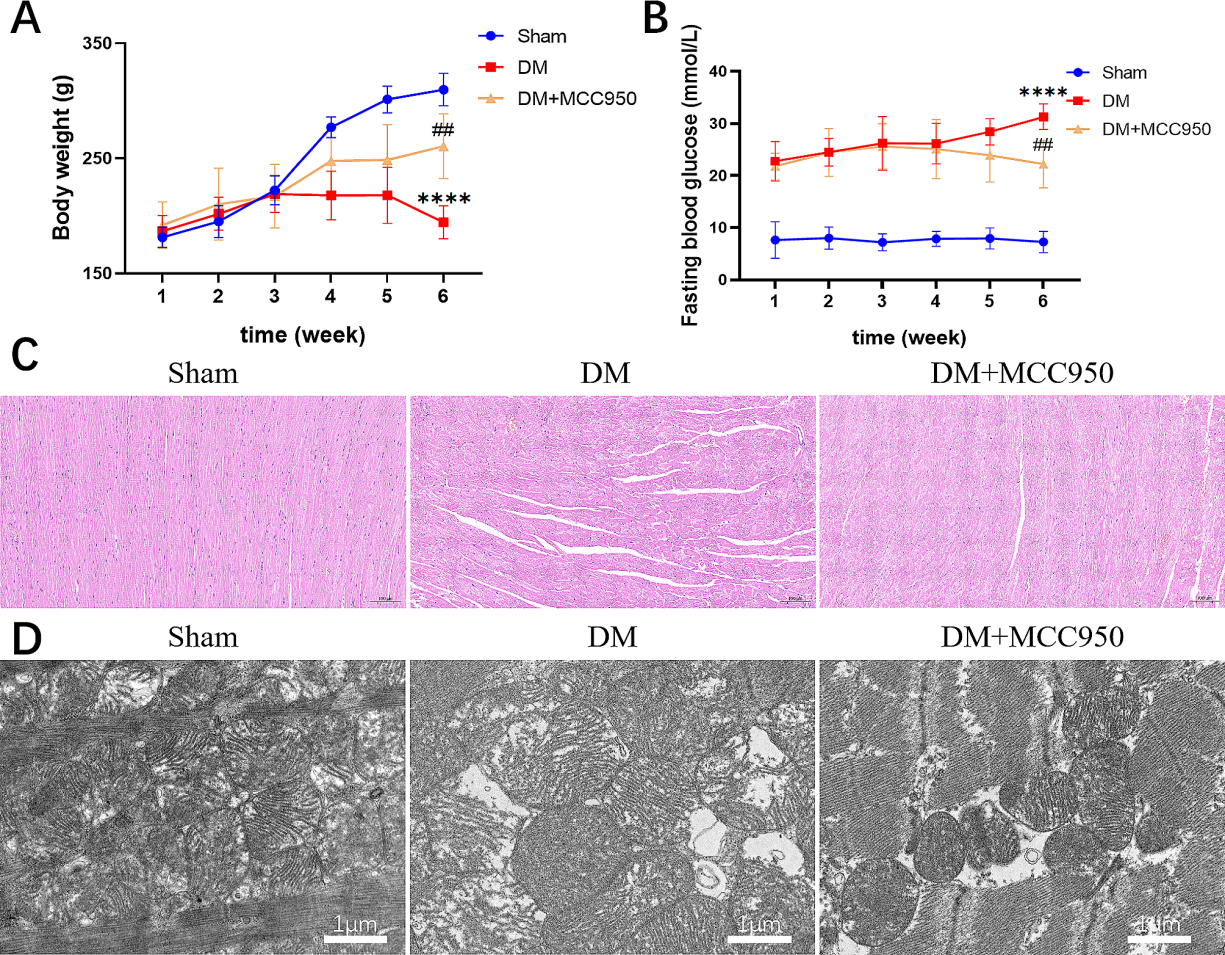
Changes of BW, FBG and Structural in myocardial organization of rats. The level of BW (**A**) and FBG (**B**) levels in each group. (mean ± SD, *n* = 6). ****P < 0.0001 vs. Sham; ^##^*P* < 0.01 vs. DM. Images of HE staining (**C**) and transmission electron microscopy (**D**) of rat myocardial tissue in each group (C: 100×, scale bar = 100 μm, D: 20,000×, scale bar = 1 μm)

**Fig. 2:**
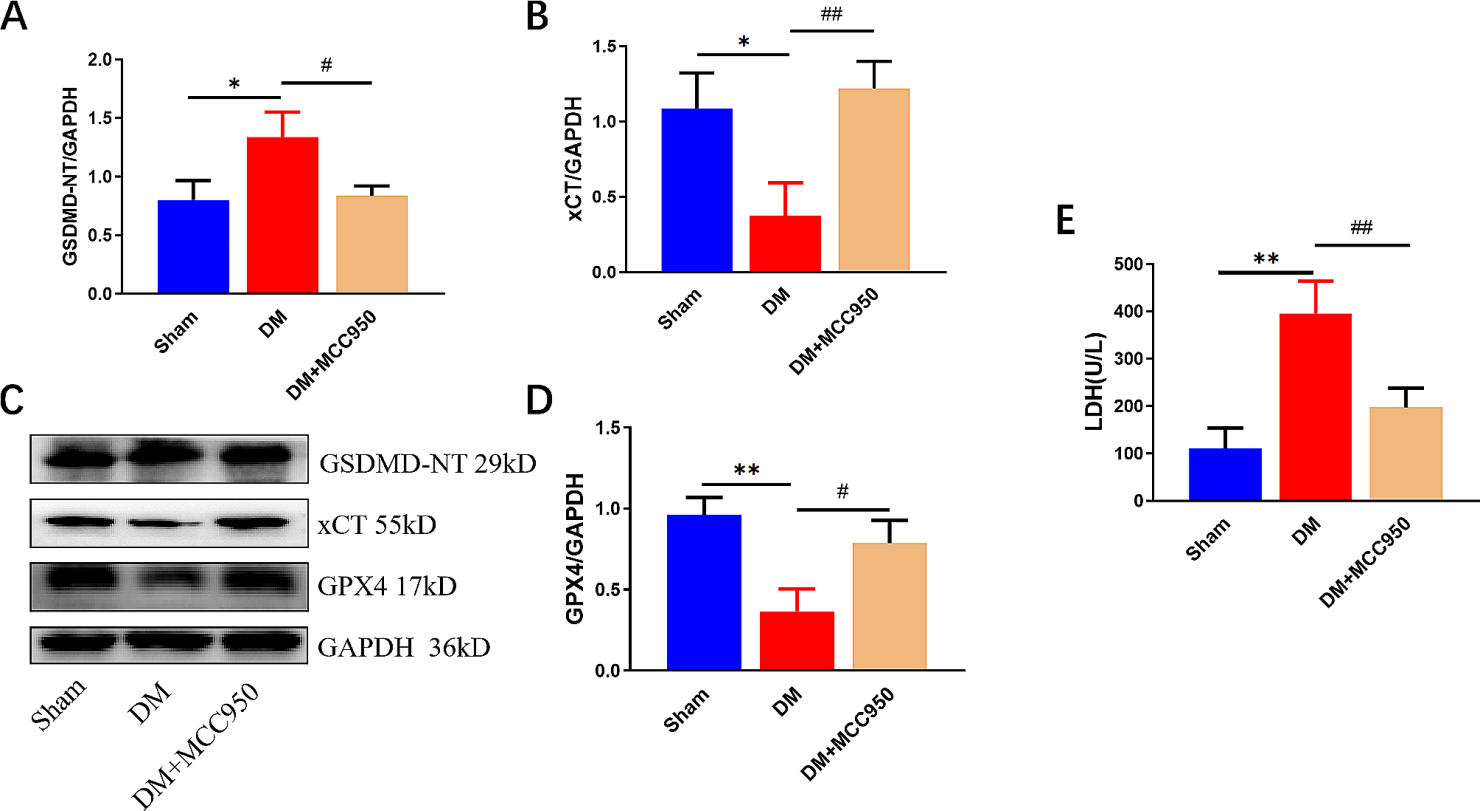
MCC950 inhibits the expressions of pyroptosis- and ferroptosis-related proteins in rat myocardial tissues and LDH levels in serum. The protein levels of xCT (**A**), GSDMD-NT (**B**), and GPX4 (**D**) in rats normalized by GAPDH levels. (**C**) Representative blots in rats. (**E**) Changes of LDH levels in serum of each group. (mean ± SD, *n* = 3). *P < 0.05, **P < 0.01 vs. Sham; ^#^*P* < 0.05, ^##^*P* < 0.01 vs. DM

MCC950, an inhibitor of NLRP3, was intraperitoneally injected into rats at 55 mg/kg twice a week. As the results shown in Fig. 1A-B, the DM group showed a gradual decrease in BW and a significant increase in FBG levels over time. Furthermore, the myocardial fibers were loosely arranged, accompanied by cardiomyocyte hypertrophy, peripheral inflammatory cell infiltration, and mitochondrial cristae were disorganized, or fused to form vacuole-like lesions, compared with the Sham group (Fig. 1C-D). the treatment with MCC950 alleviated the above symptoms. As for Fig. 2, MCC950 not only directly reduces the expression of GSDMD, the pyroptosis executor, but also affects ferroptosis -related factors, GPX4 and xCT, which are both indispensable to inhibit the occurrence of ferroptosis. Additionally, the level of LDH release in the serum of diabetic rats were effectively inhibited by MCC950 (Fig. 2E). The results above suggested that a relationship between the two forms of cell death via NLRP3 and suppress it could take on a protective effect against diabetes.

### NLRP3 knockdown attenuates HG-induced H9C2 cardiac cells injury

**Fig. 3:**
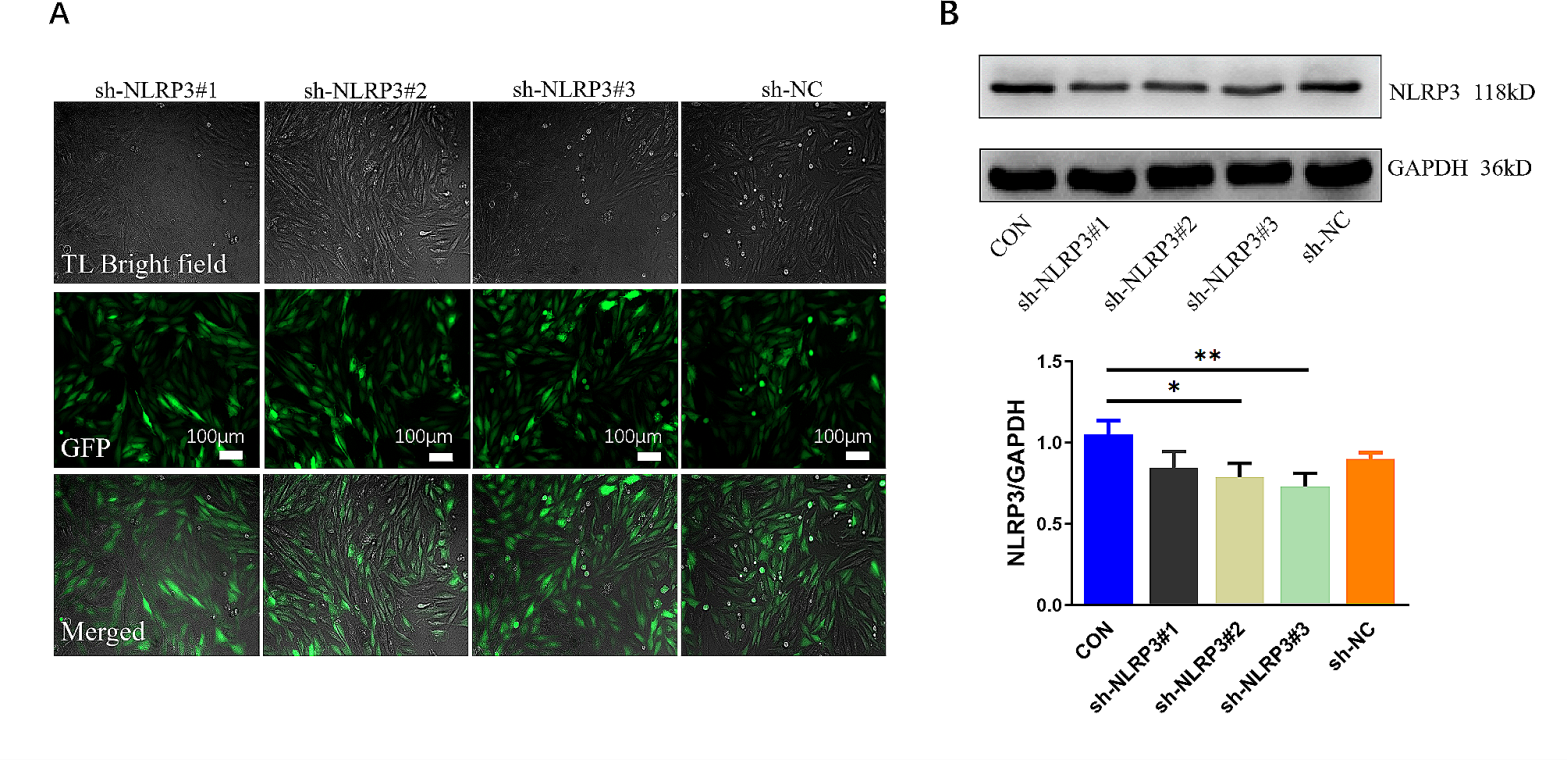
Establishment and verification of a stable sh-NLRP3 cell line. H9C2 cells were transfected with three target shRNA vectors of NLRP3 (sh-NLRP3#1, #2 and #3) and negative control vector (sh-NC). (**A**) Features of H9C2 cardiac cells under optical microscopy (100×, scale bar = 100 μm). (**B**) NLRP3 protein levels in H9C2 cardiac cells normalized by GAPDH levels. (mean ± SD, *n* = 3). *P < 0.05, **P < 0.01 vs. CON

As shown in Fig. 3, sh-NLRP3#3 minimized the NLRP3 expression in H9C2 cells although NLRP3 was verified to be knockdown in sh-NLRP3#1, #2 and #3 group, with a normal expression in the sh-NC group (Fig. 3), therefore, the sh-NLRP3#3 was used for subsequent studies.

**Fig. 4:**
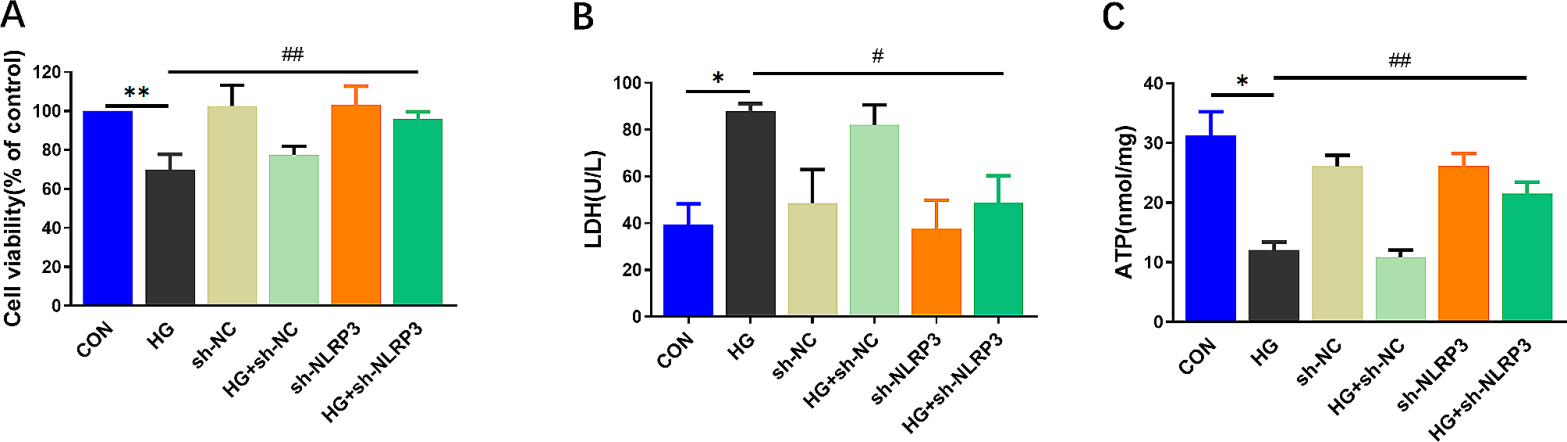
NLRP3 knockdown attenuates HG-induced H9C2 cardiac cells injury. The cell viability (**A**), LDH (**B**) and ATP (**C**) levels of H9C2 cardiac cells in the different groups. (mean ± SD, *n* = 3). *P < 0.05, **P < 0.01 vs. CON; ^#^*P* < 0.05, ^##^*P* < 0.01 vs. HG

Cell viability was detected by CCK-8 and was notably reduced after HG and HG + sh-NC treatment compared with the untreated group (Fig. 4A). However, the cell viability was increased by pre-transfection of sh-NLRP3 prior to HG compared with HG alone.

Furthermore, the results in Fig. 4B demonstrated that LDH release, which signifies cytotoxicity, was significantly increased after HG intervention, subsequently, treatment with transfection of sh-NLRP3 reduced the release of LDH.

The ATP production was assayed to evaluate the function of mitochondrial and metabolism of H9C2 cells. The concentration level of ATP was significantly reduced in HG-treated cells compared with the control group, and it was markedly increased following sh-NLRP3 transfection (Fig. 4C).

### NLRP3 knockdown inhibits HG-induced NLRP3 inflammasome related pyroptosis in H9C2 cardiac cells

**Fig. 5:**
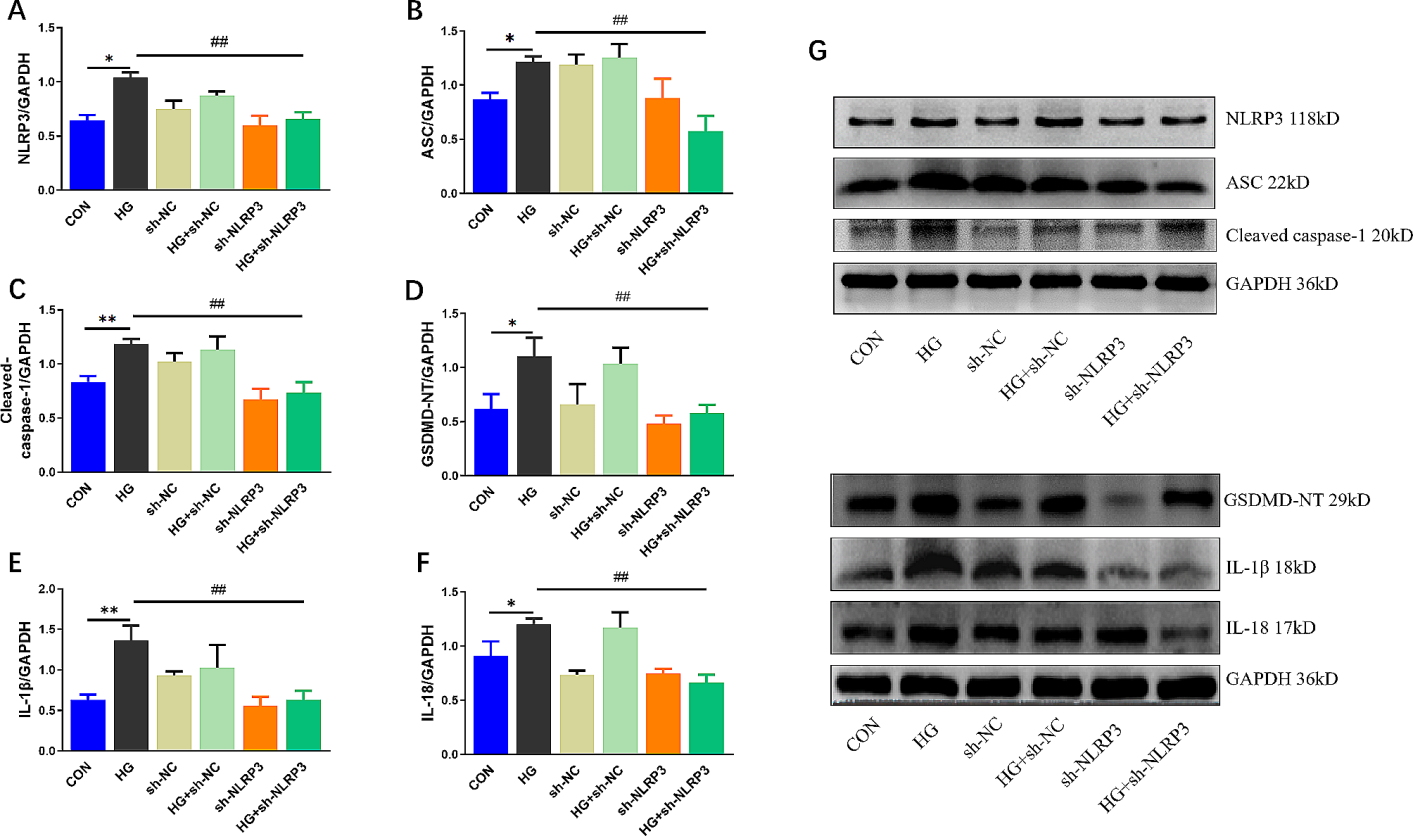
NLRP3 knockdown inhibits HG-induced NLRP3 inflammasome related pyroptosis in H9C2 cardiac cells. The protein levels of NLRP3 (**A**), ASC (**B**), Cleaved-caspase-1 (**C**), GSDMD-NT (**D**), IL-1β (**E**) and IL-18 (**F**) in H9C2 cardiac cells normalized by GAPDH levels. (**G**) Representative blots in H9C2 cardiac cells. (mean ± SD, *n* = 3). *P < 0.05, **P < 0.01 vs. CON; ^#^*P* < 0.05, ^##^*P* < 0.01 vs. HG

**Fig. 6:**
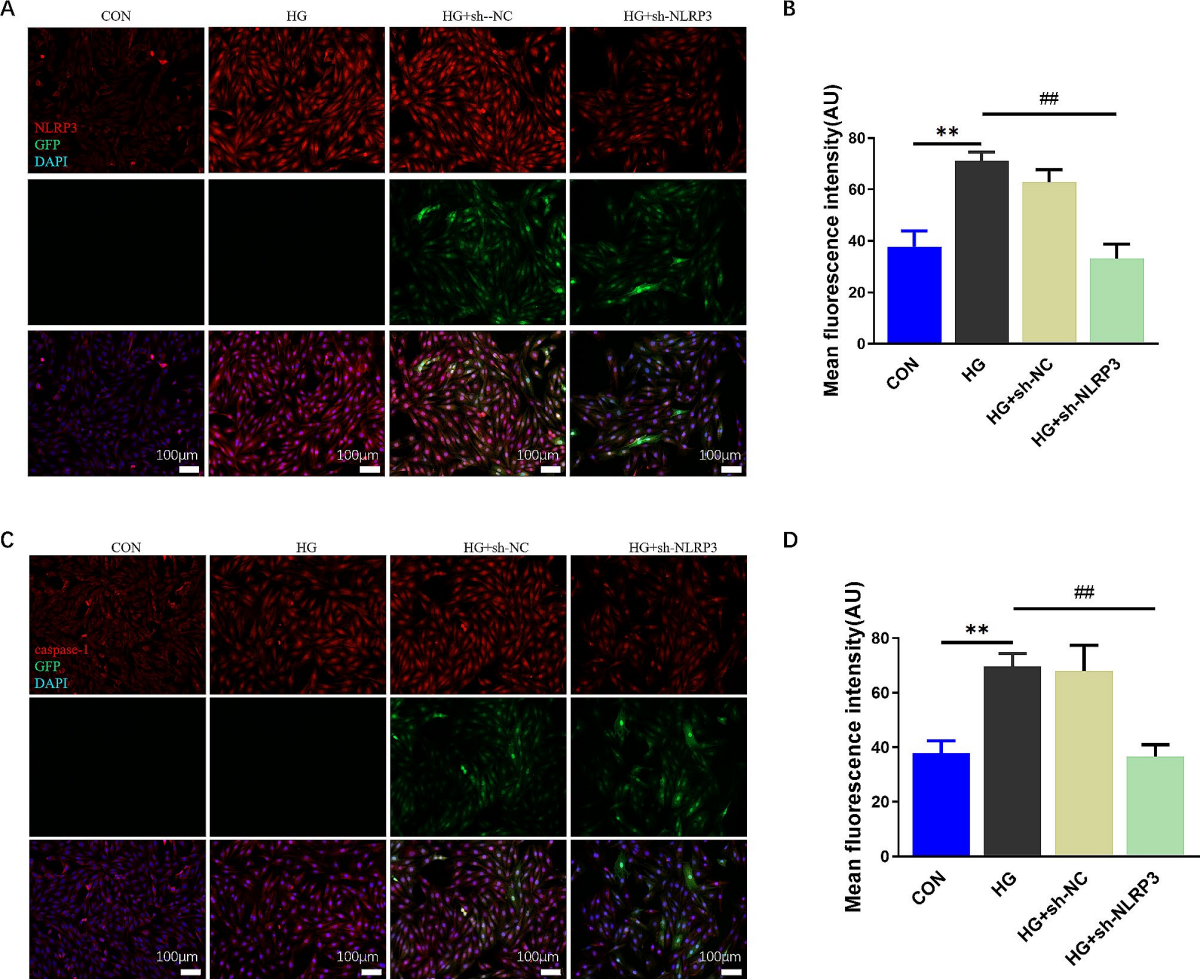
Immunofluorescence staining in H9C2 cardiac cells. Images of NLRP3 (**A**, red), caspase-1 (**C**, red), FITC (green) and DAPI (blue) immunofluorescence staining (200×, scale bar = 100 μm). NLRP3 (**B**) and caspase-1 (**D**) fluorescence intensity analysis (mean ± SD, *n* = 3). **P < 0.01 vs. CON; ^##^*P* < 0.01 vs. HG

The NLRP3 inflammasome-associated protein levels, including NLRP3, ASC, Cleaved-caspase-1 and pyroptosis associated protein GSDMD-NT level were shown to be notably higher in H9C2 cells than in controls, identical to the expressions of the inflammatory factors IL-1β and IL-18, and all of the above proteins was markedly decreased by pre-transfection of sh-NLRP3 (Fig. 5A-G). Moreover, as presented in Fig. 6, fluorescent staining of NLRP3 and caspase-1 were significantly increased after HG treatment, which was reversed by NLRP3 knockdown. These results suggested the protective effect of knocking down NLRP3 was related with down-expressions of NLRP3 inflammasome and pyroptosis on H9C2 cells.

### Promotion of mitochondrial ROS production abolishes the protective effect of NLRP3 knockdown

**Fig. 7:**
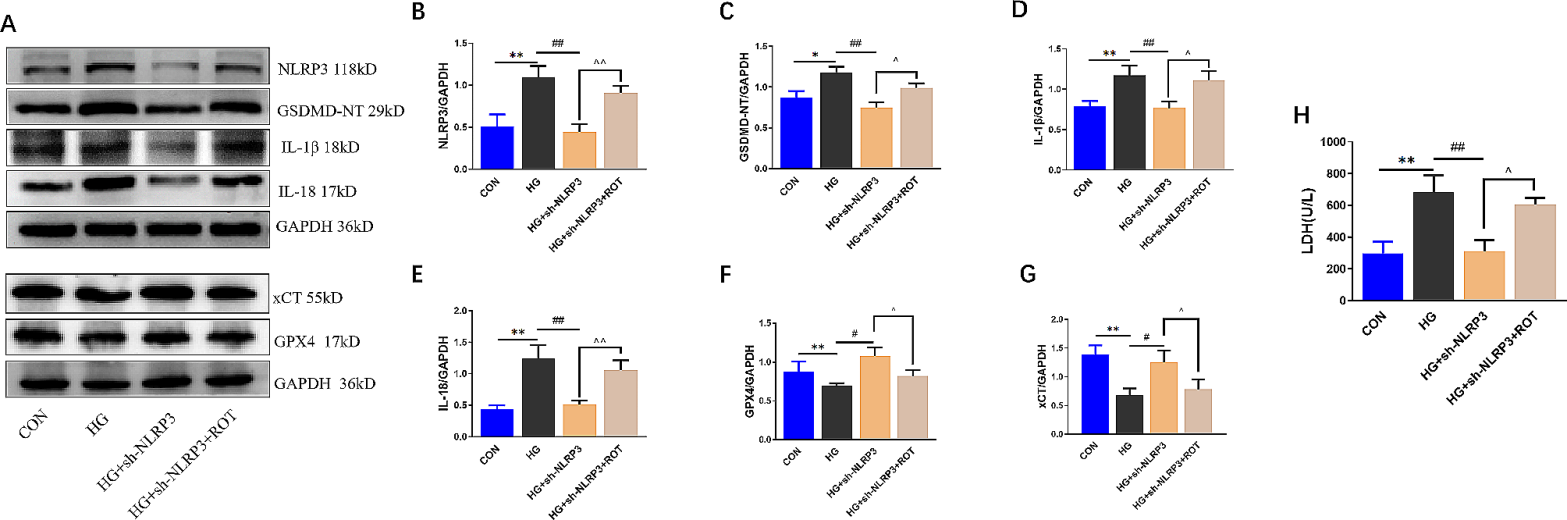
Promotion of mitochondrial ROS production abolishes the protective effect of NLRP3 knockdown. (**A**) Representative blots in H9C2 cardiac cells. The protein levels of NLRP3 (**B**), GSDMD-NT (**C**), IL-1β (**D**), IL-18 (**E**), GPX4 (**F**) and xCT (**G**) in H9C2 cardiac cells normalized by GAPDH levels. H. Changes of LDH levels in cell supernatant of each group.(mean ± SD, *n* = 3). *P < 0.05, **P < 0.01 vs. CON; ^##^*P* < 0.01 vs. HG; ^^^*P* < 0.05, ^^^^*P* < 0.01 vs. HG + sh-NLRP3

To further investigate the upstream and downstream relationship between NLRP3 and mtROS, ROT was used to promote the production of mtROS for the subsequent study. As shown by western blotting results in Fig. 7A-E, the protective effect of sh-NLRP3 on HG-induced high levels of NLRP3 inflammasome and pyroptosis-associated proteins were all reversed by ROT. Furthermore, the GPX4 and xCT that is related to ferroptosis was distinctly weakened in HG treated group, however, according to the results in Fig. 7F-G, GPX4 and xCT expression were increased significantly after promoting mtROS production. Additionally, as presented in Fig. 7H, co-treatment of sh-NLRP3 and ROT significantly elevated LDH release compared to the HG + sh-NLRP3 group. These results suggest that knocking down NLRP3 gene protects H9C2 cells from HG-induced injury, and mtROS can be located downstream of NLRP3.

**Fig. 8:**
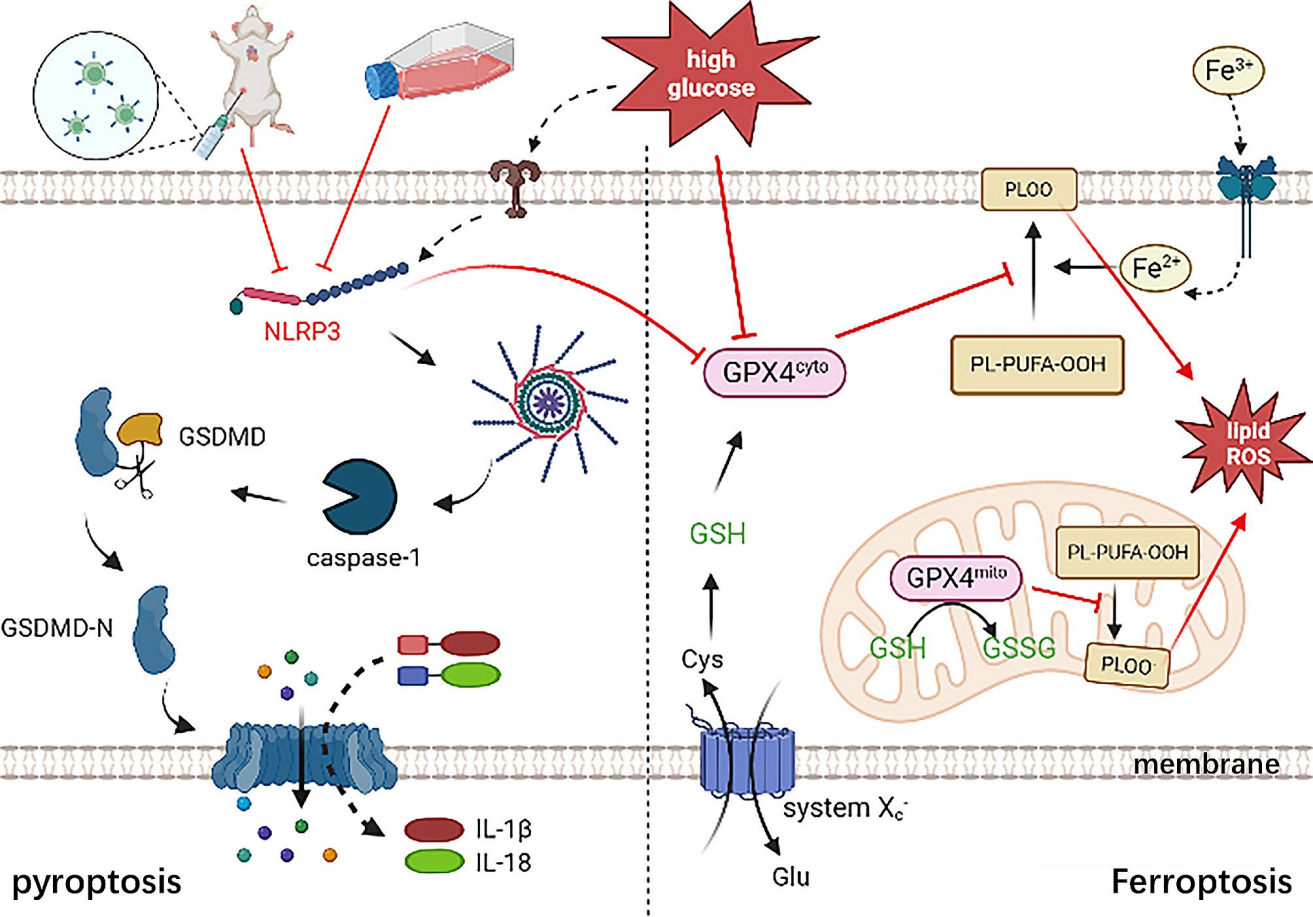
Inhibition of NLRP3 suppressed DM or high glucose-induced H9C2 cardiac cell injury through inhibiting NLRP3 inflammasome mediated pyroptosis and GPX4 mediated ferroptosis. NLRP3: The NACHT-, LRR- and PYD domains-containing protein 3 (NLRP3), GSDMD: Gasdermin D, GPX4: glutathione peroxidase 4, GSH: glutathione, PLOO: phospholipid, Cys: cystine, Glu: glutamate

## Discussion

DCM, which closely related to the high incidence of heart failure and its poor prognosis, is characterized by myocardial fibrosis and parietal stiffness. Hyperglycemia, insulin resistance and hyperinsulinemia are considered as the independent risk factors for the development of DCM and are responsible for cardiac remodeling and dysfunction. Several mechanisms have been involved in the pathogenesis of DCM, such as alterations in myocardial energy metabolism and calcium signaling. Particularly, a local rise in cytokines in cardiac cells is an important part of the development of DCM. Furthermore, HG-treated cells were widely studied in vitro to replace DCM [17, 18] and to induce pyroptosis and ferroptosis [19–22], which both are closely related to the inflammatory response. So, in the present study, STZ (55 mg/kg, i.p) were used to induce diabetic injury in vivo, simultaneously, H9C2 cells were incubated in 35 mmol/L glucose medium for 24 h to simulate the DCM model in vitro.

LDH is an important enzyme capable of affecting energy metabolism in the body, and its level in the cytoplasm reflects cell membrane permeability and cytotoxicity. LDH was found at increased levels in the heart muscle and serum in diseases such as myocardial infarction and diabetic [23, 24]. It is well documented that intracytoplasmic LDH release occurs due to the disruption of cell membrane structure caused by pyroptosis or ferroptosis [25, 26]. As the most important energy molecule, decreased ATP levels indicate impaired or reduced mitochondrial function. Decreased ATP concentrations have been shown in mitochondria isolated from the hearts of DCM rats [27]. In the present study, we found that the release of LDH were increased in DM rats, accompanied by altered expression of pyroptosis and ferroptosis-related proteins compared with the Sham group. In addition, in H9C2 cells, the NLRP3 inflammasome and pyroptosis-related proteins and inflammatory factors were significantly increased following HG treatment, accompanied by decreases in cell viability, ATP level, suggesting HG induced cardiac injury through decreasing ATP production and increasing inflammatory action, pyroptosis and ferroptosis.

NLRP3, a well-known critical component of the innate immune system, is a key step in pyroptosis. NLRP3 could be activated by various pathogen-associated molecular patterns (PAMPs) such as excess ROS and subsequently forms NLRP3-ASC-pro-caspase-1 inflammasome complex through the interaction of homotypic proteins with PYD domain and CARD domain. GSDMD-N terminal then is cleaved by activated caspase-1 and oligomerized pores in the cell membrane, releasing inflammatory factors such as IL-1β and IL-18, exacerbating the inflammatory response, which is the process of pyroptosis [28]. The protective effect of NLRP3 inhibition has been widely explored in inflammatory disorders. Blocking NLRP3 activation by MCC950, a small-molecule inhibitor of NLRP3, has been studied to attenuate the severity of autoimmune encephalomyelitis [29, 30]. Similarly, our previous study also demonstrated the protective effect of MCC950 on cardiomyocytes [31]. Mesenchymal stem cell was found to alleviate insulin resistance in T2DM rats by suppressing NLRP3 inflammasome-mediated inflammation and expression of IL-1β and IL-18 [32]. Further, numbers of studies have shown that pyroptosis is closely related to cardiovascular diseases, diabetes and diabetic complications [28, 33]. In the present study, sh-NLRP3 were transfected to H9C2 cells before HG treatment The results revealed that knockdown NLRP3 protects against HG -induced high expressions of pyroptosis-related ASC, caspase-1, GSDMD-NT, etc.

Ferroptosis is another form of programmed cell death characterized by iron-dependent lipid peroxidation and metabolic constraints. The accumulation of intracellular lipid peroxidation and ROS production exceeds the redox content maintained by glutathione (GSH), resulting in a redox imbalance and the accumulation of iron-dependent phospholipid hydroperoxide (PLOOH), which causes rapid and irreparable damage to the membrane structure of cells and organelles [34, 35] (33,34). Numerous studies have shown that ferroptosis can be endogenously counteracted by the system x_c_^−^/GSH/GPX4 axis. System X_c_^−^ is a chloride-dependent membrane cystine/glutamate antiporter, and it is a heterodimeric protein containing a light chain SLC7A11 (xCT, a 12-pass transmembrane transporter protein), and a heavy chain SLC3A2 (4F2 heavy chain, a transmembrane regulatory subunit). Compounds including erastin that interfere with system X_c_^−^ causing cysteine deprivation, GSH depletion, endoplasmic reticulum stress and cell death [36]. And GPX4, the key regulator of ferroptosis, can neutralize oxidized lipids with glutathione [37]. Moreover, GPX4 was found to be downregulated in cardiomyocytes with myocardial infarction or diabetic myocardial ischemia/reperfusion injury [26, 38]. In the present study, GPX4 and xCT protein levels were significantly down-regulated by high glucose compared to the normal cells.

Though crosstalk may occur between multiple regulated cell death forms [39, 40], the relationship between NLRP3 and ferroptosis has not yet been clarified. Surprisingly, in the present study, we found that knockdown of NLRP3 in H9C2 cardiac cells counteracted high glucose-induced low expression of GPX4 and xCT protein. Surprisingly, our results showed the same changes in the expression of GPX4 and xCT in heart tissue after the use of MCC950 to fight against diabetes in rats. The above suggesting that inhibition of NLRP3 not only inhibits pyroptosis but may also play a role in ferroptosis, but the exact mechanism needs to be further explored.

It is well known that the release of mtROS activates NLRP3 to exert inflammatory effects, nevertheless, it remains unclear whether there is a mutually regulated upstream and downstream feedback relationship between mtROS and inflammasome. ROT, as a mitochondrial electron transport inhibitor has been studied by Bavkar [41] to continue to promote ROS on top of high glucose-induced multi-organ damage such as heart, liver, lung and kidney. In fact, in our previous study, ROT was also found to counteract part of the protective effect of MCC950 [31]. Therefore, in the present study, ROT was used to promote more mtROS production in H9C2 cardiac cells on the basis of knockdown of NLRP3. The results showed that the protective effect of knockdown of NLRP3 against HG injury was reversed following by ROT treatment, and pyroptosis-related protein expression as well as LDH release were notably increased, while GPX4 was reduced. These findings indicate that promotion of mtROS release after knockdown of NLRP3 can continuously exert the damaging effects through inducing pyroptosis and ferroptosis, which tips an interactive relationship between mtROS and NLRP3, that is mtROS is well known to stimulate NLRP3 upstream, but our study found that mtROS can also play an agonistic feedback regulation on NLRP3 downstream. Then, whether mtROS can directly induced pyroptosis and ferroptosis not depending on NLRP3, or depending on other mechanisms? It needs to investigate intensively.

In summary, our findings produce the evidence that knocking down of NLRP3 effectively protect against HG-induced H9C2 cardiac cells injury by inhibiting pyroptosis and ferroptosis, further, there is a crosstalk between these two cell death forms through NLRP3 (Fig. 8). Additionally, promoting mtROS production also plays an impairing role even downstream of NLRP3. These above results provide a new idea or target point for further investigation of HG-induced H9C2 cardiac cell disorders. However, the mechanism of NLRP3, a key factor of pyroptosis, affecting ferroptosis has not been represented in this study and needs to be investigated in our next step.

### Electronic supplementary material

Below is the link to the electronic supplementary material.

Supplementary Material 1

## Data Availability

Data is provided within the manuscript or supplementary information files.

## Abbreviations

DCM: Diabetic cardiomyopathy
HG: High glucose
NLRP3: NACHT-, LRR- and PYD domains-containing protein 3
STZ: Streptozotocin
ASC: Composed of NLRP3, apoptosis-associated speck-like protein containing CARD
ROS: Reactive oxygen species
mtROS: Mitochondrial ROS
shRNA: Small hairpin RNA
GSDMD: Gasdermin D
GPX4: Glutathione peroxidase 4
ROT: Rotenone

## References

[1] Wang Y, Luo W, Han J (2020). Others. MD2 activation by direct AGE interaction drives inflammatory diabetic cardiomyopathy. Nat Commun.

[2] Zhao MX, Zhou B, Ling L (2017). Others. Salusin-beta contributes to oxidative stress and inflammation in diabetic cardiomyopathy. Cell Death Dis.

[3] Zamora M, Villena JA. Contribution of impaired insulin signaling to the Pathogenesis of Diabetic Cardiomyopathy. Int J Mol Sci. 2019;20(11). 10.3390/ijms20112833. 10.3390/ijms20112833 PMC6600234 31212580

[4] Jubaidi FF, Zainalabidin S, Mariappan V. and others. Mitochondrial Dysfunction in Diabetic Cardiomyopathy: The Possible Therapeutic Roles of Phenolic Acids. Int J Mol Sci. 2020;21(17). 10.3390/ijms21176043. 10.3390/ijms21176043 PMC7503847 32842567

[5] Chen Y, Hua Y, Li X (2020). Others. Distinct types of cell death and the implication in Diabetic Cardiomyopathy. Front Pharmacol.

[6] Wu M, Han W, Song S (2018). Others. NLRP3 deficiency ameliorates renal inflammation and fibrosis in diabetic mice. Mol Cell Endocrinol.

[7] Wan X, Xu C, Lin Y. and others. Uric acid regulates hepatic steatosis and insulin resistance through the NLRP3 inflammasome-dependent mechanism. J Hepatol. 2016;64(4):925 – 32. 10.1016/j.jhep.2015.11.022. 10.1016/j.jhep.2015.11.022 26639394

[8] Luo B, Huang F, Liu Y (2017). Others. NLRP3 inflammasome as a molecular marker in Diabetic Cardiomyopathy. Front Physiol.

[9] Yang F, Qin Y, Wang Y (2019). Others. Metformin inhibits the NLRP3 Inflammasome via AMPK/mTOR-dependent effects in Diabetic Cardiomyopathy. Int J Biol Sci.

[10] Luo B, Li B, Wang W (2014). Others. NLRP3 gene silencing ameliorates diabetic cardiomyopathy in a type 2 diabetes rat model. PLoS ONE.

[11] Kelley N, Jeltema D, Duan Y. and others. The NLRP3 Inflammasome: An Overview of Mechanisms of Activation and Regulation. Int J Mol Sci. 2019;20(13). 10.3390/ijms20133328. 10.3390/ijms20133328 PMC6651423 31284572

[12] Murphy MP (2009). How mitochondria produce reactive oxygen species. Biochem J.

[13] Wang H, Liu C, Zhao Y (2020). Others. Mitochondria regulation in ferroptosis. Eur J Cell Biol.

[14] Wu X, Zhang H, Qi W (2018). Others. Nicotine promotes atherosclerosis via ROS-NLRP3-mediated endothelial cell pyroptosis. Cell Death Dis.

[15] Wang Y, Zhao Y, Wang Z. and others. Peroxiredoxin 3 Inhibits Acetaminophen-Induced Liver Pyroptosis Through the Regulation of Mitochondrial ROS. Front Immunol. 2021;12:652782. 10.3389/fimmu.2021.652782. 10.3389/fimmu.2021.652782 PMC8155593 34054813

[16] Jelinek A, Heyder L, Daude M (2018). Others. Mitochondrial rescue prevents glutathione peroxidase-dependent ferroptosis. Free Radic Biol Med.

[17] Liu C, Han Y, Gu X (2021). Others. Paeonol promotes Opa1-mediated mitochondrial fusion via activating the CK2alpha-Stat3 pathway in diabetic cardiomyopathy. Redox Biol.

[18] Ding W, Feng H, Li WJ (2021). Others. Apocynin attenuates diabetic cardiomyopathy by suppressing ASK1-p38/JNK signaling. Eur J Pharmacol.

[19] Cao R, Fang D, Wang J (2019). Others. ALDH2 overexpression alleviates high glucose-Induced cardiotoxicity by inhibiting NLRP3 inflammasome activation. J Diabetes Res.

[20] Jing G, Wang H, Nan F (2021). Others. Naofucong ameliorates high glucose Induced hippocampal neuron Injury through suppressing P2 × 7/NLRP1/Caspase-1 pathway. Front Pharmacol.

[21] Zhang J, Qiu Q, Wang H (2021). Others. TRIM46 contributes to high glucose-induced ferroptosis and cell growth inhibition in human retinal capillary endothelial cells by facilitating GPX4 ubiquitination. Exp Cell Res.

[22] Ma H, Wang X, Zhang W (2020). Others. Melatonin suppresses Ferroptosis Induced by high glucose via activation of the Nrf2/HO-1 signaling pathway in type 2 Diabetic osteoporosis. Oxid Med Cell Longev.

[23] Yu Y, Jin L, Zhuang Y (2018). Others. Cardioprotective effect of rosuvastatin against isoproterenol-induced myocardial infarction injury in rats. Int J Mol Med.

[24] Uyar A, Abdulrahman NT (2020). A histopathological, immunohistochemical and biochemical investigation of the antidiabetic effects of the Pistacia terebinthus in diabetic rats. Biotech Histochem.

[25] Mu Y, Sun J, Li Z. and others. Activation of pyroptosis and ferroptosis is involved in the hepatotoxicity induced by polystyrene microplastics in mice. Chemosphere. 2022;291(Pt 2):132944. 10.1016/j.chemosphere.2021.132944. 10.1016/j.chemosphere.2021.132944 34793849

[26] Li W, Li W, Leng Y (2020). Others. Ferroptosis is involved in diabetes myocardial Ischemia/Reperfusion Injury through endoplasmic reticulum stress. DNA Cell Biol.

[27] Kurian AM (2020). Mitochondrial dysfunction plays a key role in the abrogation of cardioprotection by sodium hydrosulfide post-conditioning in diabetic cardiomyopathy rat heart. Naunyn Schmiedebergs Arch Pharmacol.

[28] Yu ZW, Zhang J, Li X (2020). Others. A new research hot spot: the role of NLRP3 inflammasome activation, a key step in pyroptosis, in diabetes and diabetic complications. Life Sci.

[29] Coll RC, Robertson AA, Chae JJ (2015). Others. A small-molecule inhibitor of the NLRP3 inflammasome for the treatment of inflammatory diseases. Nat Med.

[30] Hou B, Zhang Y, Liang P (2020). Others. Inhibition of the NLRP3-inflammasome prevents cognitive deficits in experimental autoimmune encephalomyelitis mice via the alteration of astrocyte phenotype. Cell Death Dis.

[31] Wang J, Liang H, Fang D. and others. [Inhibition of mitochondrial reactive oxygen species reduces high glucose-induced pyroptosis and ferroptosis in H9C2 cardiac myocytes]. Nan Fang Yi Ke Da Xue Xue Bao. 2021;41(7):980–87. 10.12122/j.issn.1673-4254.2021.07.03. 10.12122/j.issn.1673-4254.2021.07.03 PMC8329685 34308846

[32] Sun X, Hao H, Han Q. and others. Human umbilical cord-derived mesenchymal stem cells ameliorate insulin resistance by suppressing NLRP3 inflammasome-mediated inflammation in type 2 diabetes rats. Stem Cell Res Ther. 2017;8(1):241. 10.1186/s13287-017-0668-1. 10.1186/s13287-017-0668-1 PMC5667486 29096724

[33] Ji N, Qi Z, Wang Y and others., Pyroptosis. A New Regulating Mechanism in Cardiovascular Disease. J Inflamm Res. 2021;14:2647–66. 10.2147/JIR.S308177. 10.2147/JIR.S308177 PMC8235951 34188515

[34] Yang WS, Stockwell BR (2016). Ferroptosis: death by lipid peroxidation. Trends Cell Biol.

[35] Dixon SJ, Lemberg KM (2012). Lamprecht MR and others. Ferroptosis: an iron-dependent form of nonapoptotic cell death. Cell.

[36] Sato M, Kusumi R, Hamashima S. and others. The ferroptosis inducer erastin irreversibly inhibits system xc – and synergizes with cisplatin to increase cisplatin’s cytotoxicity in cancer cells. Scientific Reports. 2018;8(1). 10.1038/s41598-018-19213-4. 10.1038/s41598-018-19213-4 PMC5772355 29343855

[37] Seibt TM, Proneth B, Conrad M (2019). Role of GPX4 in ferroptosis and its pharmacological implication. Free Radic Biol Med.

[38] Park TJ, Park JH, Lee GS. and others. Quantitative proteomic analyses reveal that GPX4 downregulation during myocardial infarction contributes to ferroptosis in cardiomyocytes. Cell Death Dis. 2019;10(11):835. 10.1038/s41419-019-2061-8. 10.1038/s41419-019-2061-8 PMC6828761 31685805

[39] Tang R, Xu J, Zhang B (2020). Others. Ferroptosis, necroptosis, and pyroptosis in anticancer immunity. J Hematol Oncol.

[40] Tang D, Kang R, Berghe TV. and others. The molecular machinery of regulated cell death. Cell Res. 2019;29(5):347–64. 10.1038/s41422-019-0164-5. 10.1038/s41422-019-0164-5 PMC6796845 30948788

[41] Bavkar LN, Patil RS, Rooge SB (2019). Others. Acceleration of protein glycation by oxidative stress and comparative role of antioxidant and protein glycation inhibitor. Mol Cell Biochem.

