# Supplementary figures and images for: Effect of NLRP3 gene knockdown on pyroptosis and ferroptosis in diabetic cardiomyopathy injury
Source: BMC Cardiovasc Disord. 2024 Jul 10;24:351. doi: 10.1186/s12872-024-04010-x (PMC11234732; doi:10.1186/s12872-024-04010-x)

Fig. 2

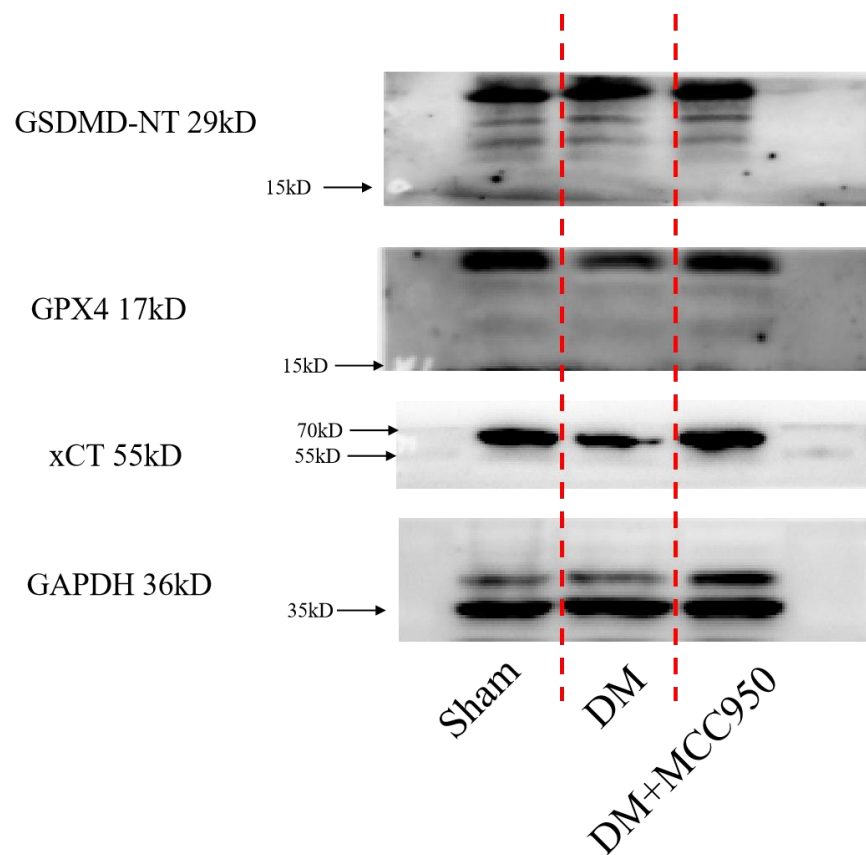

Fig. 3

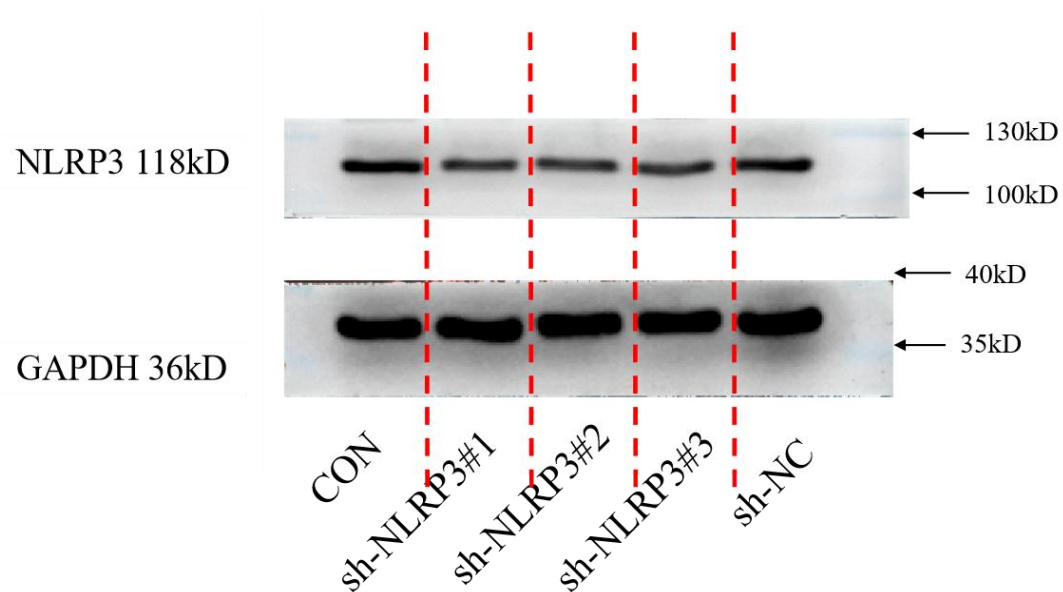

Fig. 5

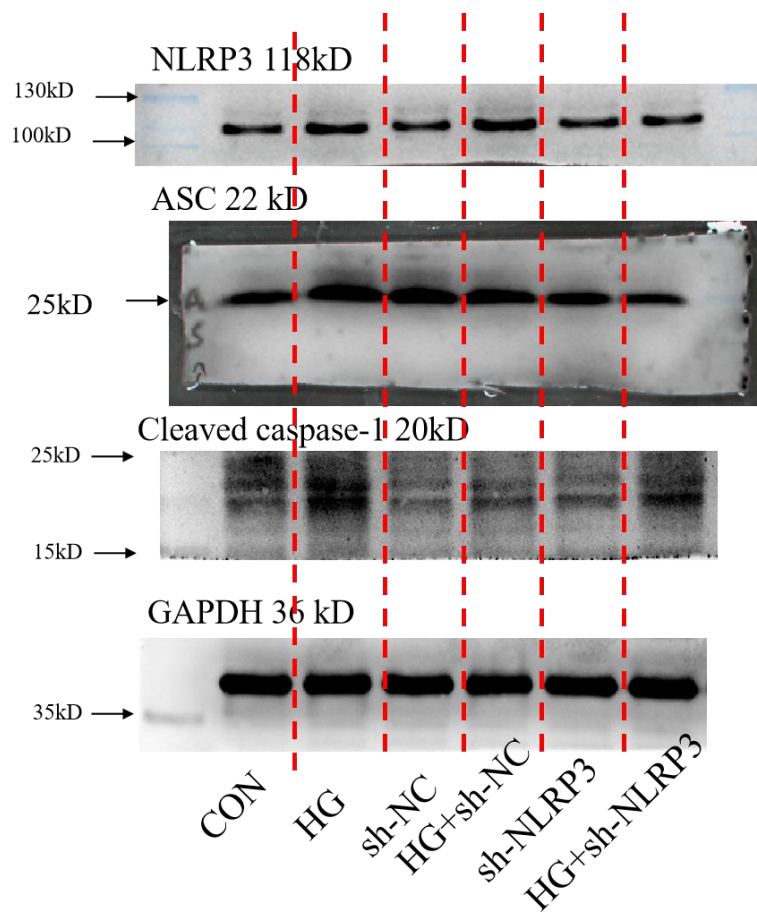

GSDMD-NT 29kD

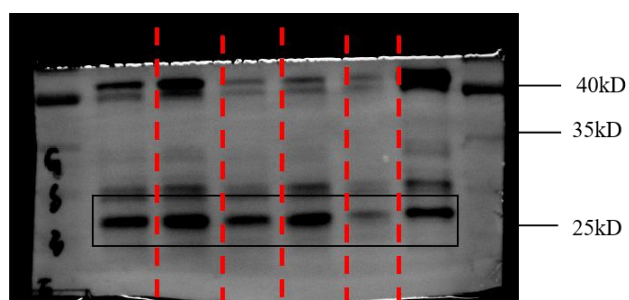

IL-1 $\beta$  18kD

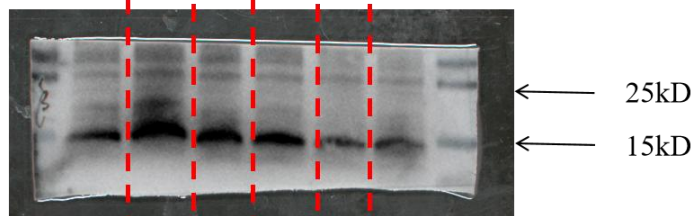

IL-18 17kD

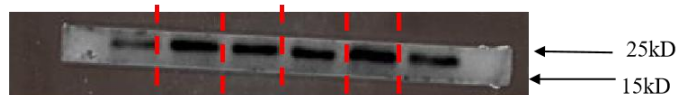

GAPDH 36kD

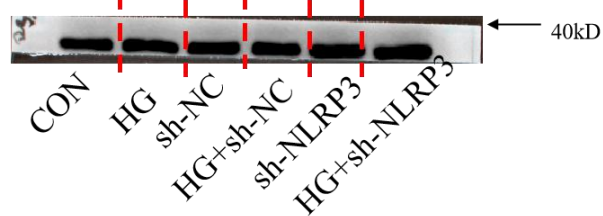

CON HG sh-NC HG+sh-NC sh-NLRP3 HG+sh-NLRP3

Fig. 7

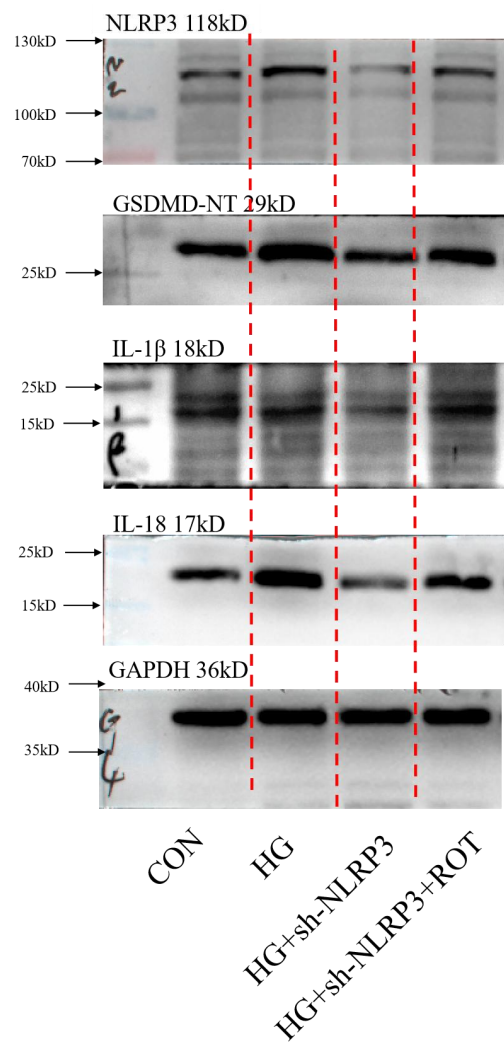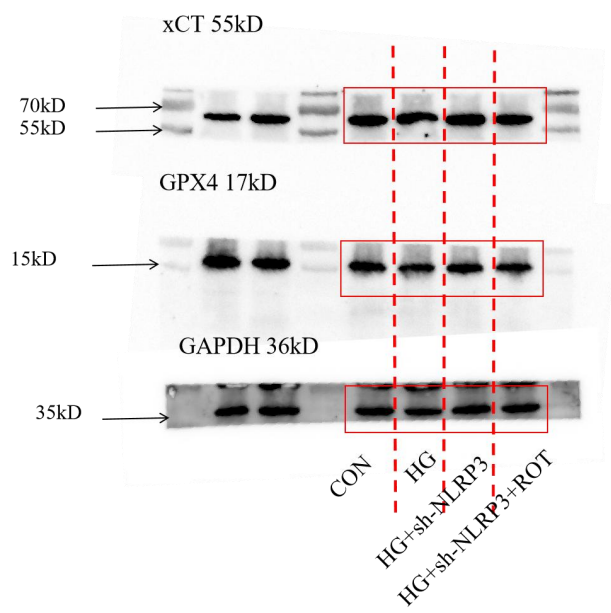

Supplement: Supplementary file 1 — Supplementary Material 1 [file 12872_2024_4010_MOESM1_ESM.pdf]
